# A New Chamber for Studying the Behavior of *Drosophila*

**DOI:** 10.1371/journal.pone.0008793

**Published:** 2010-01-27

**Authors:** Jasper C. Simon, Michael H. Dickinson

**Affiliations:** Division of Biology, California Institute of Technology, Pasadena, California, United States of America; Chiba University Center for Forensic Mental Health, Japan

## Abstract

Methods available for quickly and objectively quantifying the behavioral phenotypes of the fruit fly, *Drosophila melanogaster*, lag behind in sophistication the tools developed for manipulating their genotypes. We have developed a simple, easy-to-replicate, general-purpose experimental chamber for studying the ground-based behaviors of fruit flies. The major innovative feature of our design is that it restricts flies to a shallow volume of space, forcing all behavioral interactions to take place within a monolayer of individuals. The design lessens the frequency that flies occlude or obscure each other, limits the variability in their appearance, and promotes a greater number of flies to move throughout the center of the chamber, thereby increasing the frequency of their interactions. The new chamber design improves the quality of data collected by digital video and was conceived and designed to complement automated machine vision methodologies for studying behavior. Novel and improved methodologies for better quantifying the complex behavioral phenotypes of *Drosophila* will facilitate studies related to human disease and fundamental questions of behavioral neuroscience.

## Introduction

**Figure 1:**
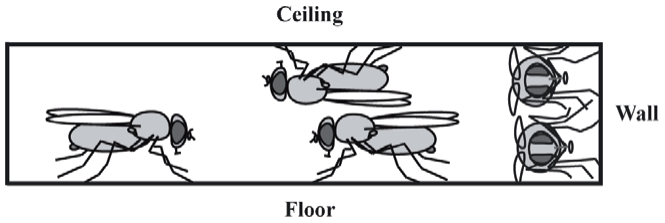
Side-view illustration of typical arrangements of flies in chambers with vertical walls. Problematic conjunctions occur when a fly clings to the ceiling, partially occluding a fly standing on the floor, and when two flies stand one above the other on the wall.

Due to the development of sophisticated genetic tools, *Drosophila* has emerged as a powerful model system for studying the causal relationships between genes, neurons, and behavior [1]–[3]. Progress in identifying such relationships is inhibited by the fact that the methods available for quantifying behavior lag in sophistication behind the tools available for manipulating gene or neuron function [4]. Machine vision offers a promising strategy for automatically tracking and measuring the behavioral phenotypes of flies [5]–[13]. However, the robustness of these automatic methodologies is highly dependent on the quality of the raw data contained within the digital movies of the flies' behavior. Conventional chambers used for studying the behaviors of flies possess several features that make the measurement and analysis of behavior difficult. For example, high ceilings permit flight, which is difficult to track using a single low-temporal resolution camera. Vertical walls in a chamber allow flies to walk up and onto the ceiling, creating a situation in which flies may overlap and obscure each other. Vertical walls also lead to significant changes in the appearance of flies as they move among the different surfaces of the floor, wall, and ceiling. These deviations in appearance can obscure identifiable features that might have been useful for detecting specific behaviors, such as the position of the fly's wings and limbs. Furthermore, cracks, corners, and vertical surfaces are attractive to flies and promote their clustering on the wall or in the periphery of the chamber. These features all result in problematic scenarios for automatic tracking methods based on a digital video stream (Fig. 1).

Here we present a new strategy for constructing experimental chambers that restrict the behavior of flies to within a monolayer. Low ceilings prevent flies from hopping or flying over each other, an acute angle formed between sloped walls and the ceiling reduces the number of flies walking onto the ceiling, and a slippery ceiling limits the duration flies may cling to the ceiling before falling to the floor. Previous methods to keep flies within a monolayer have required elaborate designs, such as water moats [14] or thermal barriers [13], and the limitation and tedium of clipping off the flies' wings. Our design lessens the probability that flies obscure each other, limits the variability in their appearance due to moving among various regions of the chamber, and promotes a greater number of flies to move throughout the center region of the arena. Flies within the new chamber can exhibit all of the behaviors normally observed in a laboratory setting, with the exception of flight. The new design helps in generating improved-quality raw data and therefore complements machine vision methodologies for automated studies of complex behavioral phenotypes of fruit flies.

## Results

### Fewer Problematic Conjunctions

**Figure 2:**
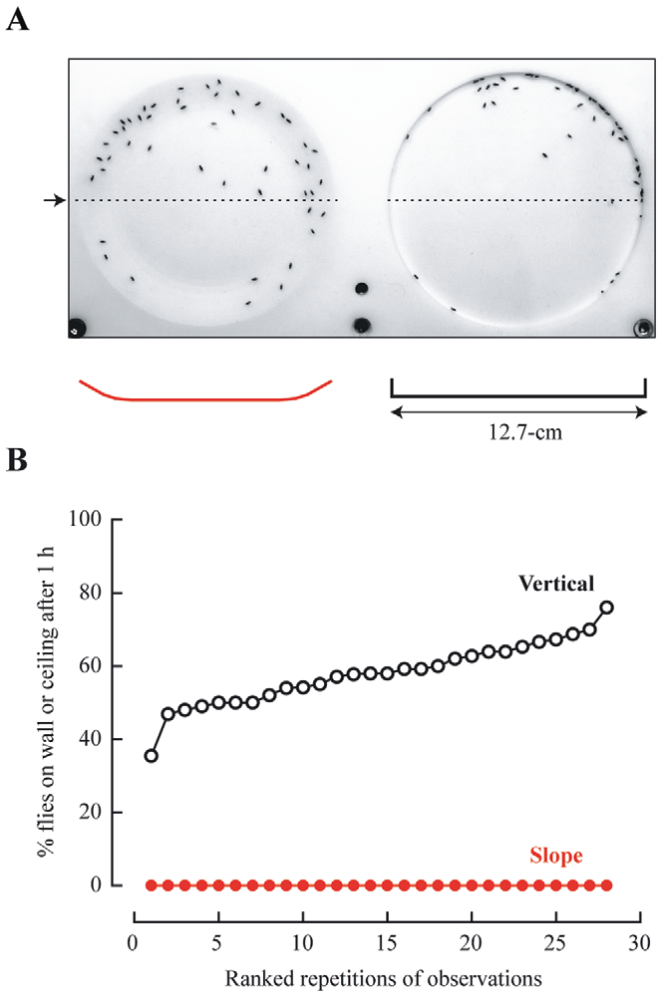
Sloped walls lessened the probability of problematic conjunctions between flies. (A) Photograph showing a typical distribution of 50 flies observed in chambers with sloped and vertical walls with associated drawings that depict the contour of the floors of the chambers along the cross section shown (arrow). (B) Comparison of the percent of problematic flies from groups of 50 individuals observed after 1 hour in chambers with sloped (red closed circles) and vertical walls (black open circles).

To compare the number of flies that have a high probability of overlapping, we introduced groups of 50 flies into chambers with our new sloped-wall design and conventional chambers with vertical walls. Aside from the shape of the wall, the chambers had comparable heights and diameters. After allowing flies to settle for 1 hour, we counted the number of problematic flies, i.e., flies residing on the walls and ceiling of each chamber (Fig. 2). For 14 days, we observed groups of flies introduced into 2 chambers with sloped walls and 2 chambers with vertical walls. As expected, chambers with sloped walls contained negligible numbers of problematic flies, whereas the percent of problematic flies in chambers with vertical walls ranged from 30% to 70% (Fig. 2B).

### Behavior Restricted to Monolayer

**Figure 3:**
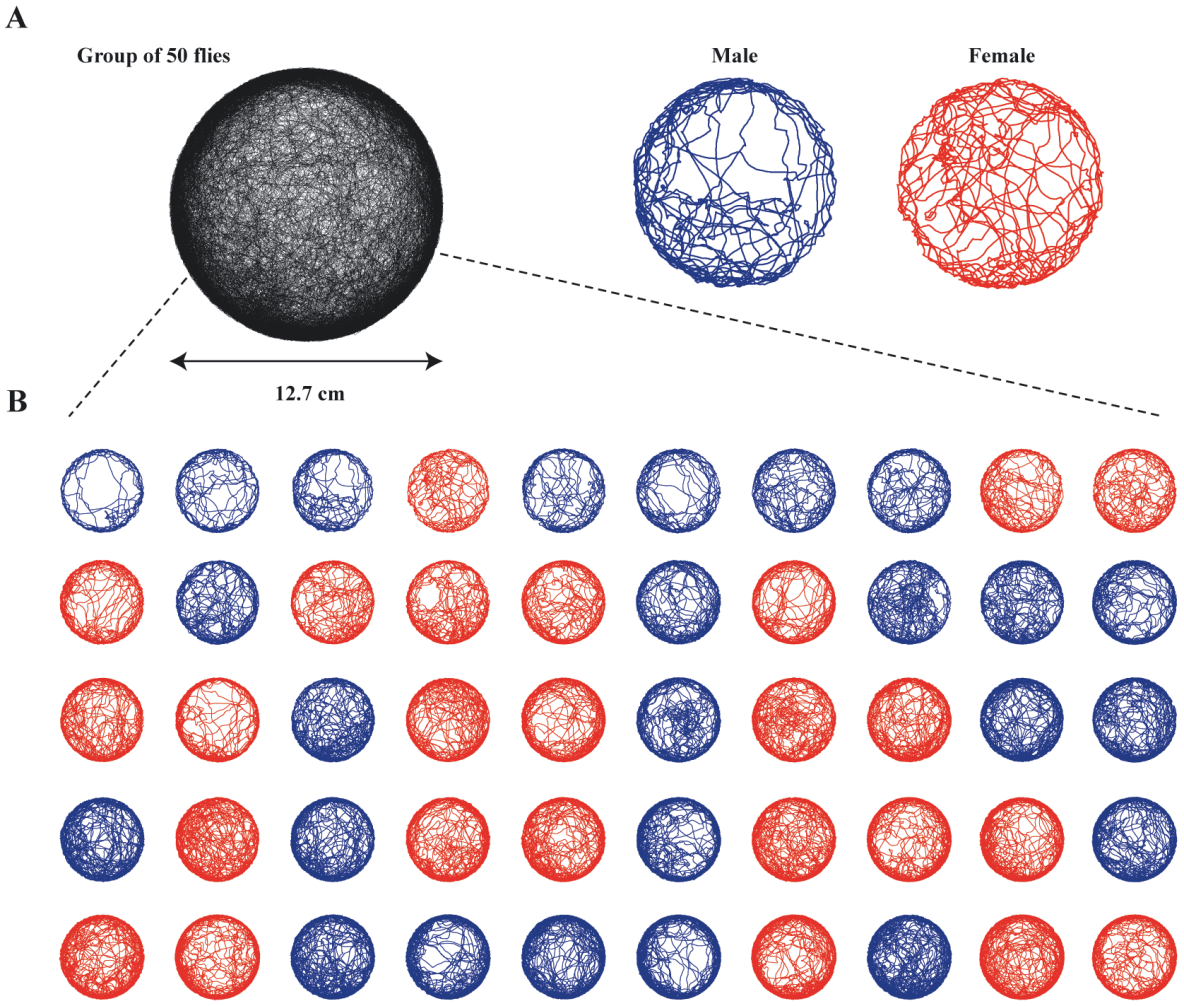
Trajectories of 50 flies moving for 10 minutes within a chamber designed with sloped walls. (A) Superposition of the individual trajectories from the group of 50 flies. (B) Individual trajectories of the 50 flies making up the group shown in **A** with individual males (blue) and females (red) sorted along rows from the shortest to the longest distance traveled (Top left to bottom right).

To illustrate how chambers with the new design complement automatic methodologies for studying behavior, we used Ctrax software designed to track and retain the identity of individuals within large groups of flies [13]. For these observations, we introduced 25 male and 25 females flies into a 12.7-cm diameter chamber with sloped walls and recorded their movements for 30 min (Fig. 3; Movie S1). Ctrax requires that the flies remain within a planar arena and not overlap. As described above, chambers designed with sloped walls prevented flies from obscuring each other by moving up the wall or onto the ceiling of the chamber. The glass ceiling on these chambers prevented flies from leaving and also allowed an unobstructed view for recording their behavior. By design, the entire chamber was uniformly backlit, creating high contrast silhouettes of the flies to facilitate the tracking of their movements and classifying their identity and gender. As indicated in Fig. 3, the Ctrax software was particularly robust when analyzing data collected in our sloped-wall chambers.

### Reduced Variability in Appearance

**Figure 4:**
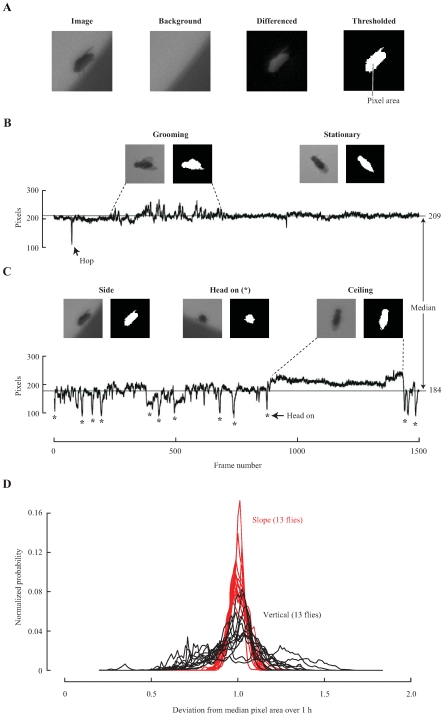
Sloped walls reduced the variability in a fly's appearance. (A) Cropped image taken from a movie that included only a small region surrounding the fly, a cropped background image from the same region of the chamber excluding the fly, a differenced image between the cropped image and the cropped background image, and a binerized representation of the difference between the images determined by a threshold. The total number of pixels from the binerized representation of the flies was calculated for each frame. (B, C) Examples using 100-second windows of movie illustrating the lower variability in the total number of pixels extracted from movies of flies recorded within the chambers with sloped walls, as compared to those with vertical walls. The median pixel area was calculated from the entire movie (gray line) and was approximately equal to when the fly was on the floor and stationary in the chambers with sloped walls. (B) Significant deviations from the median pixel area in the chamber with sloped walls corresponded to a hop (arrow) and a period when the fly was grooming (region between dashed lines). (C) Deviations in pixel area in the chamber with vertical walls were due to changes in the fly's appearance as it rotated on the wall between side and head on (astrisk) or moved from the wall onto the ceiling (region between dashed lines). (D) Normalized histogram of deviation in pixel area over the first hour of movie from flies observed in the chambers with sloped (red) and vertical walls (black). Numbers on the x-axis represent the deviation from the median pixel area, where 1 is no deviation and 0.5 and 1.5 are 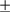 50% deviation from the median pixel number.

To compare the variability of a fly's appearance between chambers with sloped and vertical walls, we used movies recorded of single flies moving for the first hour after introducing them into the chambers [15]. After subtracting the corresponding background image, we determined the number of pixels making up a thresholded representation of the fly from each frame for each movie (Fig. 4A). For each fly we determined its median pixel value during the entire length of each 6-hour movie. We used the number of pixels from each frame over the median number of pixels from the entire 6-hour movie as a proxy measure for the deviation in a fly's appearance (Fig. 4B, C). From direct observation of movies, we observed that much of the deviation in appearance in chambers with sloped walls was due to changes in the fly's behavior, including short flights, hops, grooming, various wing movements, and changes in typical walking posture. In addition to the deviation due to these changes in behavior, large deviations resulted from changes in the fly's profile when it moved among the floor, wall, and ceiling in chambers with vertical walls. To illustrate that there was less variability in a fly's appearance in chambers with sloped walls, we compared the deviation in appearance for 26 flies, introducing 13 flies into each chamber (Fig. 4D). The results indicate that variation in pixel area is much lower in the sloped-wall chambers.

### Decreased Measurement Errors

**Figure 5:**
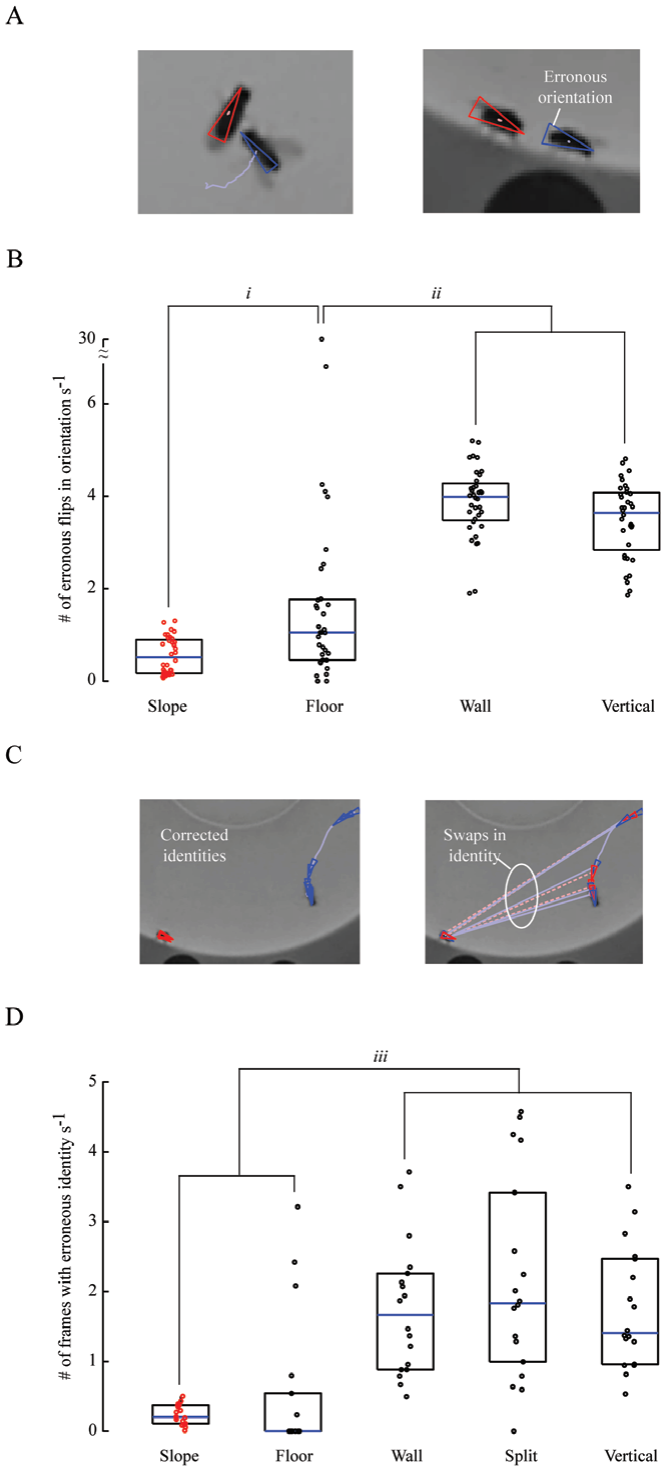
Example of movie images of males courting females, including corresponding errors in automatic classifications by body orientation and identity. (A) Examples of movie images with body orientations annotated (triangle apex denotes the position of the fly's head) that were extracted with the CADABRA software system. (B) Error rates of body orientations calculated for individual flies from movies recorded for males courting females within the chambers with sloped (red) and vertical (black) walls. Error rates from the chambers with vertical walls were decomposed into erroneous flips in orientation during periods when individual flies were either on the floor or on the wall. Medians (blue) and 25 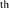 and 75 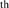 percentiles are shown (black box). (C) Examples of movie images with identity annotated while males (blue) courted females (red). Trajectories represent the location of flies for the past 30 frames (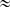 1 s). Swaps in identity are denoted by the discontinuities in the trajectories and changes in color between triangles representing past locations of flies, and therefore can be compared to a movie image that has been corrected (left). (D) Error rates for the classification of identity between pairs of male and females from movies recorded within the chambers with sloped (red) and vertical (black) walls. Error rates from the chambers with vertical walls were decomposed into swaps in identity during periods when both flies were on the floor, both on the wall, and split with one fly on the floor and the second on the wall. Medians (blue) and 25 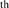 and 75 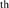 percentiles are shown (black open box). The rate of errors were similar between *Wall* vs. *Split*, Mann-Whitney U, p = 0.628; *Wall* vs. *Vertical*, Mann-Whitney U, p = 0.864; and *Split* vs. *Vertical*, Mann-Whitney U, p = 0.521.

CADABRA, a recently developed method for automatically measuring social interactions, bases its classifications for specific behaviors on changes in the relative position between flies [12]. This method then fine-tunes the classifications and determines the *detection* of specific behaviors by correlating the measured positions to changes in the flies' appearance, i.e., patterns of wing postures or measures of relative body length and width. For such a strategy to work, it is critical that measurements of body orientation and the identity of flies are correct. Here we used the outputs from CADABRA to illustrate that the vertical walls found in conventional chambers increase the number of measurement errors for body orientation and fly identity, undoubtedly contributing to missed and mischaracterized social interactions. We analyzed 36 movies of single males courting single females up to the onset of copulation or up to 20 minutes, whichever came first. Half of these movies were recorded in a new sloped-wall chamber and half were recorded in a conventional chamber with vertical walls. We measured the number of erroneous flips in body orientation by comparing the output of CADABRA to an estimate of body orientation based on a global optimization from all frames of a fly's trajectory from each movie sequence, part of the error-correcting capacities of the Ctrax software [13]. This optimization simultaneously finds the head-tail assignment for all frames such that (1) the change in the orientation between consecutive frames is small and (2) the velocity direction and orientation of a fly match the frames in which the fly is walking. We used the difference between the automatic measurement from CADABRA and the corrected estimate as a metric for the number of erroneous flips in orientation. We also estimated the number of frames containing erroneous swaps in identities between flies, by setting a classification threshold in which both flies had an identical change in the distance of their positions that was greater than 1.5 mm within a single frame. We based this estimation on the changes in their positions between consecutive frames that were also measured automatically with CADABRA. Using these metrics, we compared the number of erroneous flips in body orientation per second for individual flies and the number of frames with identities swapped per second between pairs of flies. We observed that the rate of erroneous orientation and frames containing erroneous identities were significantly less in sloped chambers than in conventional chambers (Orientation: Mann-Whitney U, p<0.0001 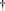; Identity: Mann-Whitney U, p<0.0001 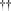; Fig. 5; Movie S2, Movie S3).

To further illustrate that it was the *vertical* walls in the conventional chambers that increased the number of measurement errors, we compared body orientations and swaps in identity when the flies were either both on the wall, both on the floor, split with one fly on the wall and the second fly on the floor, or measured for both flies irrespective of where they were throughout the chamber. The rate of erroneous orientation calculated when both flies were on the floor of conventional chambers was intermediate between the lesser rate observed for flies found throughout the sloped-wall chambers and the greater rate when both flies were on the vertical walls of conventional chambers (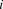 Mann-Whitney U, p = 0.012 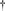; 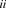 Mann-Whitney U, p<0.0001 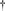; Fig. 5B). The higher rate of erroneous orientation observed when both flies were on the the vertical wall was comparable to the higher rate observed when flies were found throughout the vertical chamber (Mann-Whitney U, p = 0.312 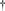, Bonferroni correction for multiple comparisons, 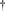 n = 36 flies for each comparison). The rate of erroneous identities for flies on the floor of conventional chambers was comparable to the rates in sloped chambers (Mann-Whitney U, p = 0.153 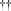). These rates were also significantly less than the rate of erroneous identities whether both or just one of the flies were on the wall of conventional chambers, and if flies were found through the chamber (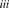 Mann-Whitney U, p<0.0001 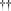; Fig. 5D). The rate of frames with erroneous identity was similar for all comparisons that included at least one fly on the vertical wall 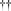 (Results from statistical analyses are within the figure legend; 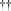 n = 18 pairs of flies for each comparison; Fig. 5D). The classification of a fly's location between the wall and floor was made based on its x, y position. Flies equal to or less than 2 mm from the periphery of the chamber were considered to be residing on the wall. From these results, it is clear that the presence of a vertical wall introduces additional variability in the appearance of flies, increasing the frequency of error in basic measures such as body orientation and identity, and undoubtedly would lead to poorer classifications and detections of behavior observed among interacting flies.

### Flies Spend Less Time in Periphery

**Figure 6:**
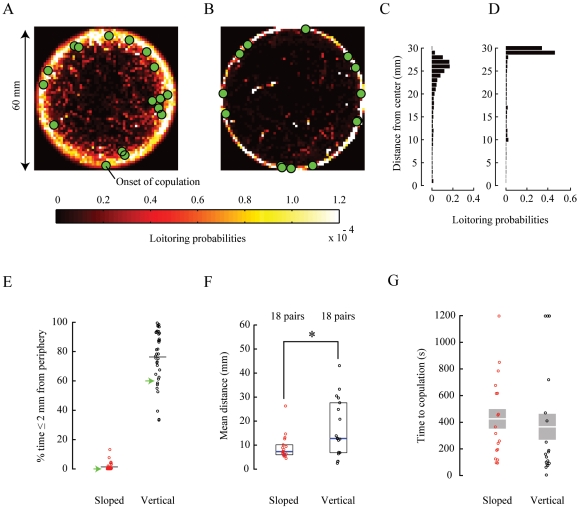
Pairs of males and females in the chambers with sloped walls spend less time near the periphery of the chamber and spend more time near each other. Normalized loitering probability prior to copulation and the locations when courting flies began copulating for (A) 18 pairs of flies in the chambers with sloped walls and (B) 18 pairs of flies in the chambers with vertical walls. Collective loitering probability normalized by area in 30 concentric regions for flies in the chambers with (C) sloped and (D) vertical walls. Concentric annuli making up the regions were 1 mm thick. (E) Collective mean percent total time (gray lines) and percent total time for individuals from pairs of flies spent near and on the wall in the chambers with sloped (red) and vertical (black) walls. Percentage of pairs of flies beginning to copulate on and near walls is also denoted (green arrowheads). (F) Collective medians (blue lines) of the average distance between pairs of flies prior to copulation in the chambers with sloped (red) and vertical (black) walls. The top and bottom of the boxes represent 25 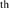 and 75 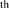 percentiles (black open box). (G) Collective means (white lines) and average copulation latencies for pairs of flies in the chambers with sloped (red) and vertical (black) walls. The top and bottom of the boxes represent 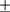 s.e.m. from collective means (gray filled box).

To quantify and compare the amount of time flies loitered in various regions in the chambers and also to observe if sloped walls might increase the interaction between flies, we introduced pairs of virgin males and virgin females into 7.0-cm diameter chambers. We then monitored their courtship until the onset of copulation or for 20 minutes, whichever came first. Flies introduced into chambers with sloped walls spent less time near the walls than in chambers with vertical walls. This difference was apparent immediately and could be seen in the trajectories of individuals making up the male-female pairs (Fig. 6A–D). This difference was also apparent in the trajectories from individuals making up large groups (Fig. S1A–C) and in the trajectories of single flies (Fig. S1E–J). We observed that flies moving near the extreme periphery of the chamber, less than or equal to 2 mm from the vertical wall, were nearly always walking on the wall. Moreover, flies moving toward vertical walls nearly always moved onto the walls and were also less often observed returning back to the chamber floor. This resulted in the flies spending the majority of their time on the wall (Fig. 6E).

The specifics of how and when the flies moved onto the wall contributed to the variability in their courtship. When females moved onto the wall first, males did not always immediately follow them, but instead sometimes spent a significant amount of time searching for the females on the chamber floor. Other times both flies moved up and onto the wall, and if this happened, usually the female would slow and stop. Once the female became stationary, typically the male would then find her, court quickly, and copulate. Alternatively, the male might move along the wall in the opposite direction and then spend a significant amount of time moving back and forth on the wall until he found her again. In several of the trials, the male would then not find the female within the observation period. In contrast, courtship was fairly uniform in chambers with sloped walls. Upon locating a potential mate, the male would court her without distraction from the geometry of the chamber until successful copulation (Movie S4). Consequently, courting pairs of flies in chambers with sloped walls were, on average, closer to each other as compared to flies in chambers with vertical walls (Fig. 6F; *Mann-Whitney U, p = 0.038, 1-tailed). However, mean 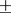 s.e.m. courtship latency, i.e., the time measured from when we released flies into the chamber until the onset of copulation, was comparable between chambers (Fig. 6G; Sloped walls, 425.5 s 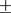 73.1 s; Vertical walls, 369.3 s 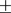 98.9 s; T-test, p = 0.629). Finally, in addition to partitioning the space used by courting pairs, vertical walls also significantly affected the quality of the flies' behavior, increasing the frequency of erratic hops and movements among the floor, wall, and ceiling (Movie S5, Movie S6).

## Discussion

We have developed a general-purpose experimental chamber that can be used for studying the locomotor behavior of single flies, interactions between pairs of flies, and the complex social interaction of individual flies behaving within large groups. The new chamber design restricts the movement of flies to a planar arena and limits variability in their appearance, without inhibiting the behaviors they typically display within a laboratory setting. The new design does not require the use of a thermal barrier [13] nor a water moat [14]. More importantly, the new design does not require clipping off the flies' wings, a manipulation that consequently inhibits a significant mode of communication during courtship or bouts of aggression. We believe the new chamber design should be complementary to a variety of methodologies designed to analyze movies from an overhead viewing angle. Moreover, the design provides a simple alternative to the more complicated machine vision methodologies that are required if cameras can view flies from difference poses.

The height of the chamber must be within critical range, but within this range, height may be tailored to fit the needs of a particular study. We have tracked the movement of flies in chambers with heights ranging from 1.8 mm to 4.5 mm. The advantage of the shorter chambers was a decrease in the frequency of overlapping flies, thereby limiting the effort required for correcting tracking errors. The trade off was that the shorter chambers restricted the repertoire of behaviors displayed by flies. For example, low chamber heights inhibit copulation [16]. In prior studies, the range of chamber heights that have been used has varied from 3 mm to 6.35 mm for studies of courtship [16]–[19] and from 11 mm to 120 mm for studies of aggression [10], [12], [20], [21]. We found chambers with a height of 3.5 mm allowed most, if not all, of the behaviors carried out between flies. (See supplementary movies illustrating various courtship and aggressive behaviors recorded from the new chamber design that may be automatically monitored with current machine vision methodologies: Movie S7–S24.) The 3.5-mm height of the chambers used within this report was optimized for *Drosophila melanogaster*, but may be easily adjusted for studying smaller and larger species of fruit flies, or even other insects.

The slope of the chamber wall was more critical than its height, but might also be adjusted. Specifically, chamber walls made more shallow than the 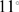 slope used here should further decrease the distance between flies. However, chambers developed with more shallow slopes will also restrict the useable space near the periphery of the chamber. It is worth noting that chambers designed with linear-sloped walls worked as well as the sigmoid-linear sloped walls described here (Fig. S2). Finally, we have tested chambers possessing diameters ranging from 30 mm to 300 mm. There does not seem to be an upper bound on the diameter of the chamber; eventually the size of the chamber will be bounded by the resolution of the camera system used.

The rapidly increasing development of new molecular tools for dissecting the genes and neural circuits regulating the behaviors of these flies has led to a recent surge in the machine vision tools that automatically monitor their complex behavioral phenotypes [8]–[13]. To make progress on the difficult task of automatically quantifying the complex social behavior of these flies, the developers of these new methodologies have focused on tracking, classifying, and quantifying behaviors. Next-generation methodologies that build upon these strategies will bundle together the key components of these methodological advances to provide a powerful tool for quantitative descriptions of the phenotypes of this genetic model organism. By restricting the movements of flies to a planar arena, limiting their profiles to a single viewing pose, and keeping flies from clustering in the periphery of the arena, we believe that the chamber design we have described within this report will make the task of automatically quantifying the complex behavior of flies significantly easier.

## Materials and Methods

### Animal Rearing, Housing, and Handling

We performed experiments on 4- to 6-day-old adult fruit flies, *Drosophila melanogaster* (Meigen), from two laboratory colonies. The first colony descended from a wild-caught population of 200 isofemales and has been used in our laboratory for approximately 15 years. The second colony was from a laboratory stock of Canton-S (CS) from the laboratory of Martin Heisenberg. We used flies from the CS colony for the observations of courtship and aggression and used the natural isolate for all other observations. We maintained fly stocks at 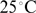 and at 40% relative humidity on Lewis food medium in standard 250 mL bottles [22], on a 16 h: 8 h light-dark photoperiod. The light-on phase started at 7AM PST. Transitions between light and dark were immediate. Replicate observations were run at the same time each day over several days, and we ran trials during either the morning or evening activity peak. We collected flies from culture bottles and housed them at a density of 50 flies per vial overnight in standard 10 mL *Drosophila* vials on food, and observed their behavior the next day. For the observation of individuals from a group of 50 flies, the morning of the day that we were to monitor their behavior, we housed 25 male and 25 female together in standard 10 mL *Drosophila* vials containing only agar in order to deprive them of food, but not water, for 7 hours prior to their observation. For our observations of courting and fighting pairs, we collected virgins <7 hours post-eclosion. We isolated males individually and housed 15 females collectively in vials containing food for 4 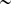 5 days before monitoring their behavior. Each day we wiped down chambers with ethanol and allowed chambers to dry for 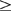 15 min. To help with counting and sorting, we immobilized flies by cooling them to 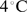 on a Peltier stage (Marlow Industries, Inc., Dallas, Texas, USA). We used a mouth pipette to introduce flies into chambers.

### Chamber Design with Sloped Walls

**Figure 7:**
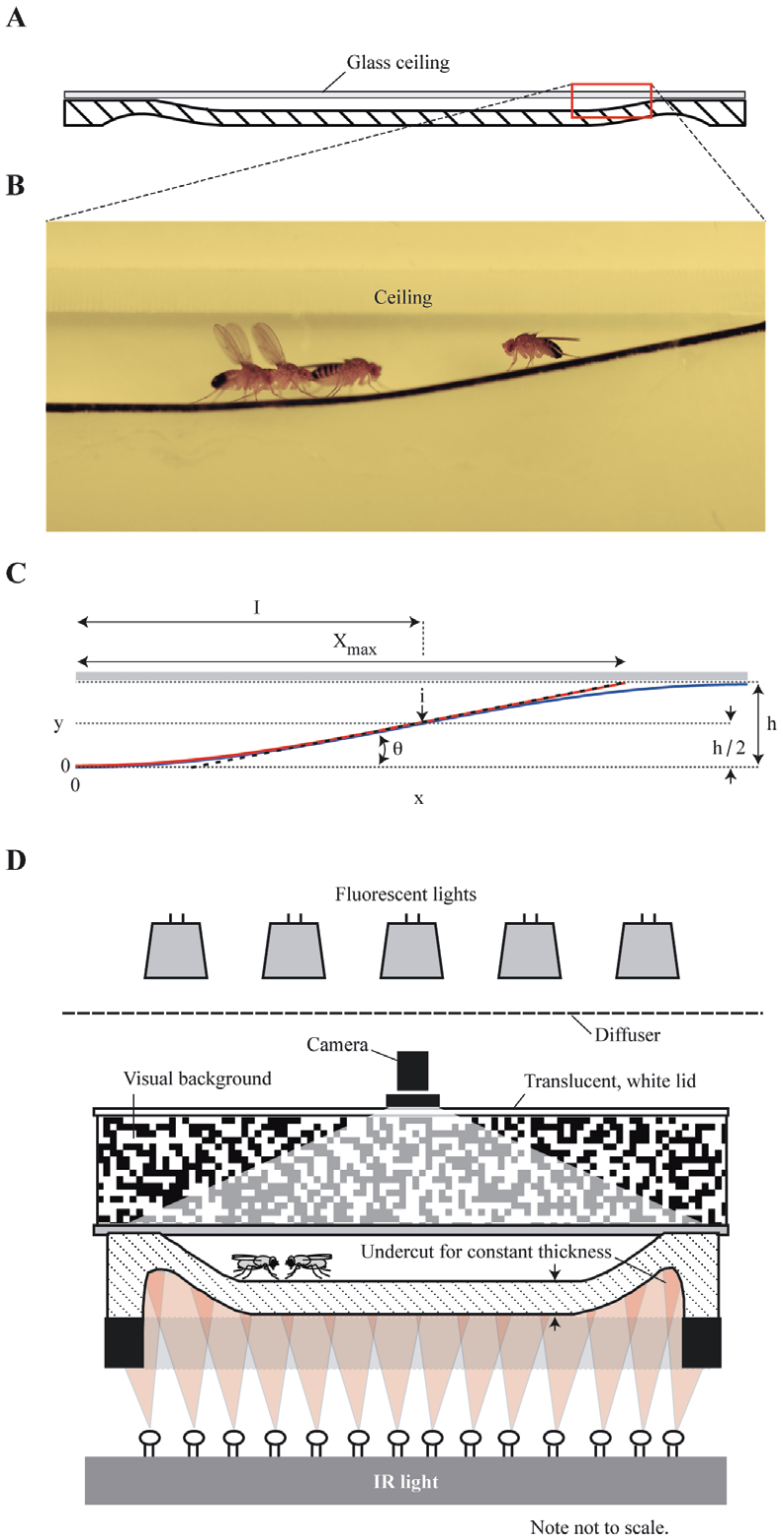
Drawings and photograph illustrating the new experimental chamber design and setup. (A) Drawing from the side of the experimental chambers, highlighting the sloped wall of the new chamber design (red box) possessing a severely acute interior angle that prevents flies from moving up the wall and onto the ceiling. The ceiling of the chambers was made of glass coated with silicone paint to limit the frequency and duration flies could cling to its surface. (B) Photograph from a cross section wedge of the chamber showing that the height of the chambers provided sufficient room for flies to carry out their normal range of locomotor behaviors. (C) Technical drawing for the profile of the slope that is described within the text. The red line depicts the slope, the blue line represents the profile of the sigmoid curve near the ceiling that was not used in making the slope, and the dashed line denotes the line tangent to the sigmoid that was matched to linear segment of the slope. (D) Drawing from the side illustrating the experimental setup. Chambers were illuminated with standard fluorescent lights projecting through a screen and a cylinder with a lid made of translucent paper. The behavior of the flies was recorded with a camera mounted above the chambers. Chambers were mounted on an aluminum base to help prevent warping and to hold the chamber above lights used for backlighting.

The key feature of the new experimental chamber design is that its walls are not vertical with square corners, as has been typical in past studies, but they were gently sloped (Fig. 7). The gently sloping walls intersected with the ceiling forming an acute interior angle that effectively deterred flies from moving onto the ceiling. Occasionally flies did move onto the ceiling, mostly as a result of jumps and flights. We have found that a ceiling made from glass coated with Sigmacote (Sigma-Aldrich), a silicone paint, provided a clear, but slippery surface that flies had difficultly clinging to. In chambers with a coated ceiling, most flies that did move onto the ceiling slipped off and fell back to floor. We have determined that a gradual slope with an angle of 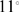, as measured from the horizontal floor, worked well in chambers with a 3.5 mm high ceiling for studying many behaviors (Fig. 7B). To remove the obtuse edge between the floor and the base of the walls, we designed the walls to have a smooth profile. The cross-sectional profile of the walls was made up of two segments, the first half a sigmoid and the second a straight line (Fig. 7C). The piecewise continuous function that specifes the height of the wall as a function of horizontal distance, 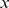, is:
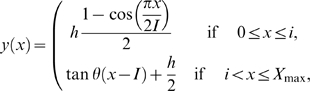
(1)where 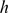 is the full height of the chamber; 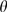 is the angle of slope; 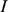 is the distance from the end of where the floor was horizontal to where the sigmoid and the straight segments join, at 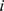, and is halfway up the height of the chamber:
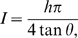
(2)and 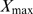 is the width of the slope from its base at the floor to where it meets the ceiling of the chamber:
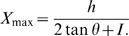
(3)


The design of the sloped walls removed the discontinuity between the floor and walls, and also eliminated the unused space that was too shallow for flies to enter if the profile of the wall followed the sigmoid to the ceiling. To keep a constant thickness for ideal backlighting, we removed material from the underside of the chamber (Fig. 7A, D). The shape of this undercut followed the curvature of the chamber floor. For the chambers discussed within this study, we machined floors to have a constant thickness of 5 mm. We observed the movement of groups and single flies within 12.7-cm diameter chambers. For the observations of courtship and aggression, we used chambers with a 7.0 cm diameter. Holes providing access to food and water were machined into the floors of chambers and used as needed (Fig. S3). To compare the behavior of flies in chambers with sloped walls to conventional chambers, we machined “control” chambers of comparable height and with comparable diameters that had vertical walls. We manufactured chambers from opaque, white Delrin (McMaster-Carr), which is easily machined, has good chemical resistance, and diffuses light, making it ideal for backlighting. We mounted chambers on base plates made from thick aluminum to insure that the chambers maintained their shape.

### Experimental Setup

To provide a visual stimulus, we surrounded the chamber described above with a paper cylinder (Fig. 7D). This paper was printed with a random checkerboard pattern with 50% black squares and 50% white squares. We capped the cylinder with an annulus cut from plain white paper so the camera lens could peer through. The cylinder and lid were backlit by an array of fluorescent lights (GE helical 26W 120 VAC 60 Hz 370 mA) and standard fluorescent room lights with a 120 Hz flicker, both shining through a projection screen (Gerriets International OPERA creamy white). The result was diffuse light creating a luminance of 75 lux at the center of the chamber. We used the visual stimulus only for observing the groups of 50 flies. Without the visual stimulus, the luminance was 500 lux. We used a 12×12-inch array of 850 nm LEDs (12×12 850 nm IR lighting unit, Illumination Control, Inc., Quincy, MA) mounted underneath the chambers for backlighting. We recorded the movements of the flies with a camera mounted from above [15]. The movements of the group of 50 flies were recorded at 20 frames per second (fps) using a 1280×1024-pixel firewire camera (Basler A622f), equipped with an 8 mm lens (Pentax). The single flies were recorded at 15 fps using a 1280×1024-pixel firewire camera (PointGray IEEE-1394), equipped with a 12 mm lens (Pentax). An infrared pass filter was placed in front of the camera to block stray light. For observing pairs of courting and aggressive flies, we used a (Sony DCR-HC38) camcorder and recorded the behavior at 30 fps as was done previously [12]. In these recordings, we backlit the chamber with visible light (Cold-cathode fluorescent backlight J58-332 8×12, Edmund Optics, Barrington, NJ).

### Machine Vision Methodologies

For the analysis of the behavior of individuals from groups of flies, we used Ctrx [13], a general-purpose system designed for tracking the individual positions and orientations of a large number of flies simultaneously. This system can be adapted to different laboratory setups and comes with software for detecting a suite of typical behavior exhibited by flies. For tracking and measuring the changes in the appearance of single flies over long durations, we used additional software developed in our laboratory. Flytrax [15] records a spatially cropped image that includes only a small region surrounding a single fly, its x,y position, and orientation from each frame. With this software, we reconstructed a high spatial and temporal representation of a fly's locomotor movement that cross-indexed each frame to its original movie. From the cropped images, we extracted the measures of the fly's appearance with custom code written in Matlab (Mathworks, Natick, MA). For monitoring and analyzing the interactions between male-female pairs, we used measurements of x, y locations, change in position, and body heading using the CADABRA system [12].

### Data and Statistical Analysis

All output measurements from the various machine vision methodologies were analyzed with custom software in Matlab. Statistical analyses were carried out using SPSS (SPSS, Inc., Chicago, IL).

### Supplementary Movies

We have included short digital movies of typical behaviors displayed by males during courtship and aggression that we believe could readily be classified from a single, top down viewing angle (Movie S7, S8, S9, S10, S11, S12, S13, S14, S15, S16, S17, S18, S19, S20, S21, S22, S23, and S24). All of these movies were recorded at 30 fps from chambers designed with sloped walls. The examples of aggression come from five 30-minute movies recording the behaviors displayed by pairs of males around a patch of food, as in [12]. The height of the ceiling for these chambers was 3.5 mm and the diameter was 7.0 cm. Frame numbers (red) are shown for all movies and can be used to determine if and when the movie was slowed down or sped up by a factor of 4, a step we took to help illustrate the behaviors.

## Supporting Information

Figure S1 Even without an attractive vertical wall, flies spend a significant amount of their time near the periphery of a chamber. Representative 0.5 hour trajectories from (A) individual flies in sloped and single flies in chambers with (E) sloped and (H) vertical walls. (B) Normalized, collective transit probability over 0.5 hour for 50 individual flies moving within a group in a chamber with sloped walls. Normalized, collective transit probability over 6 hours for 13 single flies moving in chambers with (F) sloped or (I) vertical walls. (C, G, and J) Collective transit probability normalized by area in 63 concentric regions for individual and single flies. (D) Concentric annuli making up the regions were 1 mm thick.
(0.61 MB TIF)

Figure S2 Chambers designed with linear sloped walls are comparable to the chambers designed with sigmoid-linear walls. (A) Side profile of linear slope along denoted cross section (asterisk). Obtuse corners between the wall and floor (arrowheads). (B) Superposition of the individual trajectories for 25 flies (gray) with the trajectories of two flies chosen randomly and highlighted (red and black lines).
(3.55 MB TIF)

Figure S3 Drawings of various chambers designed for studying social behavior. (A) Side view drawings of chambers designed with a plug used for courtship assays and solid resource used for observations of aggression and the conventional chamber with vertical walls possessing comparable dimensions to chambers with sloped walls. (B) Corresponding top view drawings of chambers shown in A. (C) Alternative chamber designs that could be used for providing an evenly distributed solid resource or a liquid resource from a localized spot.
(5.46 MB TIF)

Movie S1 Annotated 2-minute movie clip emphasizing the encounters made by a focal male as it moves within a group of 50 flies. The first 10 seconds of the movie show 50 un-annotated flies moving throughout the new chamber described within the body of the manuscript. The next 20 seconds show the classified identities for the same 50 flies. The following 10 seconds show the flies classified by gender (males in blue; females in red). At 40 seconds into the movie the identity and movement of the focal male is highlighted (green) within the full field image and the corresponding enlarged, spatially cropped image (center). Soon thereafter, the first fly of five encounters is highlighted (top). The movie then continues emphasizing each encounter by slowing down and highlighting the encounters within the enlarged images (clockwise from top to bottom); the encounters are color-coded within the full-field and enlarged images (with females in magenta; with males in cyan).
(23.60 MB AVI)

Movie S2 Annotated 30-second movie clip of a male courting a female in the new chamber described within this study. The body orientation (triangle apex denotes the position of the fly's head), identities of the male (blue) and female (red), and their trajectories for ∼1 second are shown.
(2.18 MB AVI)

Movie S3 Annotated 30-second movie clip of a male courting a female in the conventional chamber with vertical walls described within the body of this manuscript. The body orientation (triangle apex denotes the position of the fly's head), identities of the male (blue) and female (red), and their trajectories for ∼1 second are shown.
(2.70 MB AVI)

Movie S4 Two-minute movie showing the typically uniform behavior of a male courting a female leading to the onset of copulation within the new chamber described in the body of the manuscript.
(2.59 MB AVI)

Movie S5 Two-minute movie showing the typically more erratic behavior of a male courting a female leading to the onset of copulation within the conventional chamber with vertical walls described in the body of the manuscript.
(3.27 MB AVI)

Movie S6 Two-minute movie showing the typically more erratic behavior of a male courting a female leading to the onset of copulation within the conventional chamber with vertical walls described in the body of the manuscript.
(3.49 MB AVI)

Movie S7 Short movie clip 1 showing *wing extension* by males to females.
(2.19 MB AVI)

Movie S8 Short movie clip 2 showing *wing extension* by males to females.
(0.80 MB AVI)

Movie S9 Short movie clip 3 showing *wing extension* by males to females.
(0.80 MB AVI)

Movie S10 Short movie clip 1 showing *circling* by males around females.
(0.68 MB AVI)

Movie S11 Short movie clip 2 showing *circling* by males around females.
(0.68 MB AVI)

Movie S12 Short movie clip 3 showing *circling* by males around females.
(0.37 MB AVI)

Movie S13 Short movie clip 1 showing the *onset of copulation*.
(2.00 MB AVI)

Movie S14 Short movie clip 2 showing the *onset of copulation*.
(0.62 MB AVI)

Movie S15 Short movie clip 3 showing the *onset of copulation*.
(0.39 MB AVI)

Movie S16 Short movie clip 1 showing *guarding* and *charging* between pairs of males.
(1.13 MB AVI)

Movie S17 Short movie clip 2 showing *guarding* and *charging* between pairs of males.
(1.51 MB AVI)

Movie S18 Short movie clip 3 showing *guarding* and *charging* between pairs of males.
(1.30 MB AVI)

Movie S19 Short movie clip 1 showing *wing threat* between pairs of males.
(0.59 MB AVI)

Movie S20 Short movie clip 2 showing *wing threat* between pairs of males.
(1.13 MB AVI)

Movie S21 Short movie clip 3 showing *wing threat* between pairs of males.
(1.15 MB AVI)

Movie S22 Short movie clip 1 showing *lunges* and *head butting* between pairs of males.
(0.65 MB AVI)

Movie S23 Short movie clip 2 showing *lunges* and *head butting* between pairs of males.
(0.37 MB AVI)

Movie S24 Short movie clip 3 showing *lunges* and *head butting* between pairs of males.
(0.48 MB AVI)
