# Supplementary figures and images for: A New Chamber for Studying the Behavior of Drosophila
Source: PLoS One. 2010 Jan 27;5(1):e8793. doi: 10.1371/journal.pone.0008793 (PMC2811731; doi:10.1371/journal.pone.0008793)

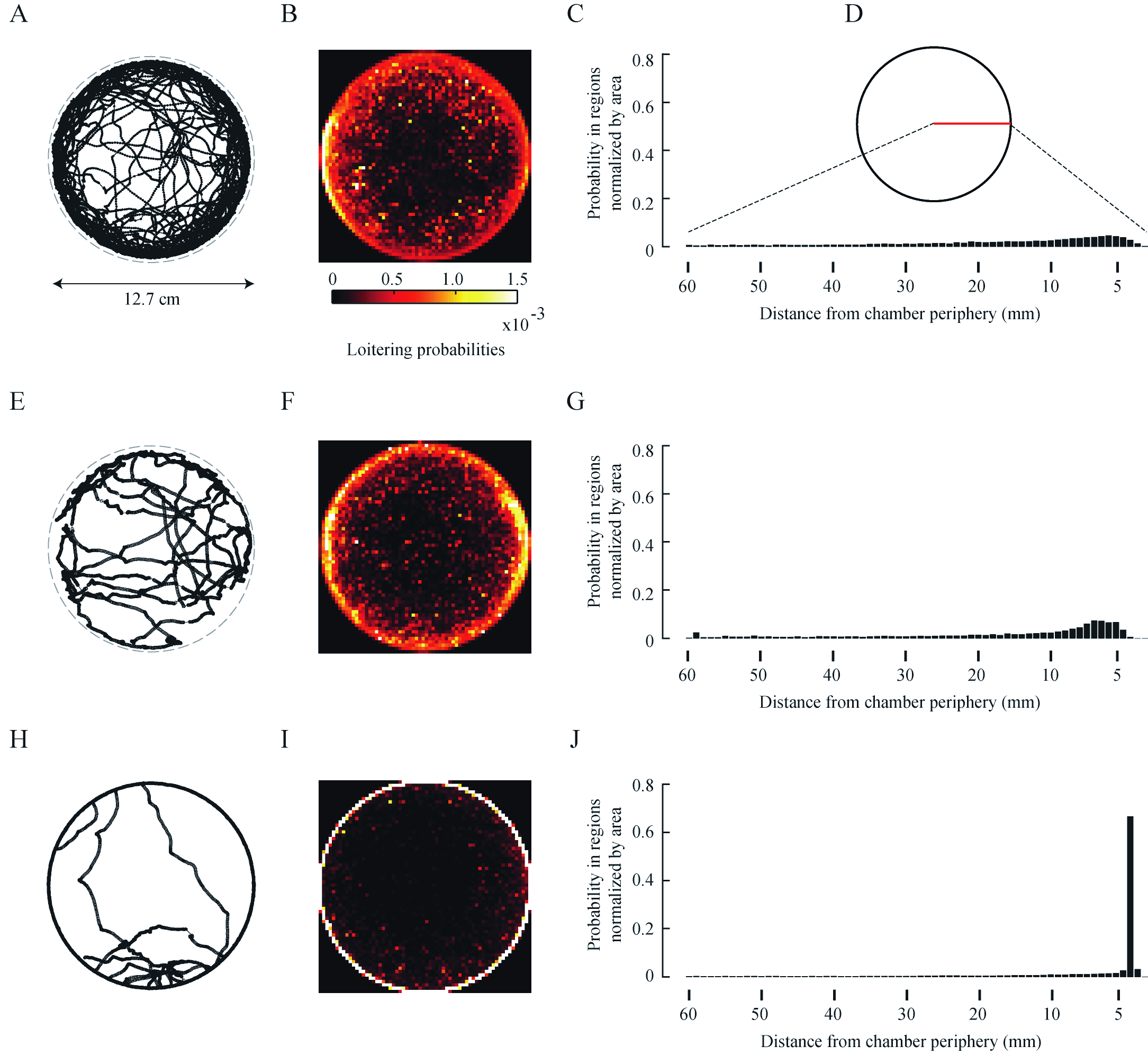

Supplement: Figure S1 — Even without an attractive vertical wall, flies spend a significant amount of their time near the periphery of a chamber. Representative 0.5 hour trajectories from (A) individual flies in sloped and single flies in chambers with (E) sloped and (H) vertical walls. (B) Normalized, collective transit probability over 0.5 hour for 50 individual flies moving within a group in a chamber with sloped walls. Normalized, collective transit probability over 6 hours for 13 single flies moving in chambers with (F) sloped or (I) vertical walls. (C, G, and J) Collective transit probability normalized by area in 63 concentric regions for individual and single flies. (D) Concentric annuli making up the regions were 1 mm thick. (0.61 MB TIF) [file pone.0008793.s001.tif]

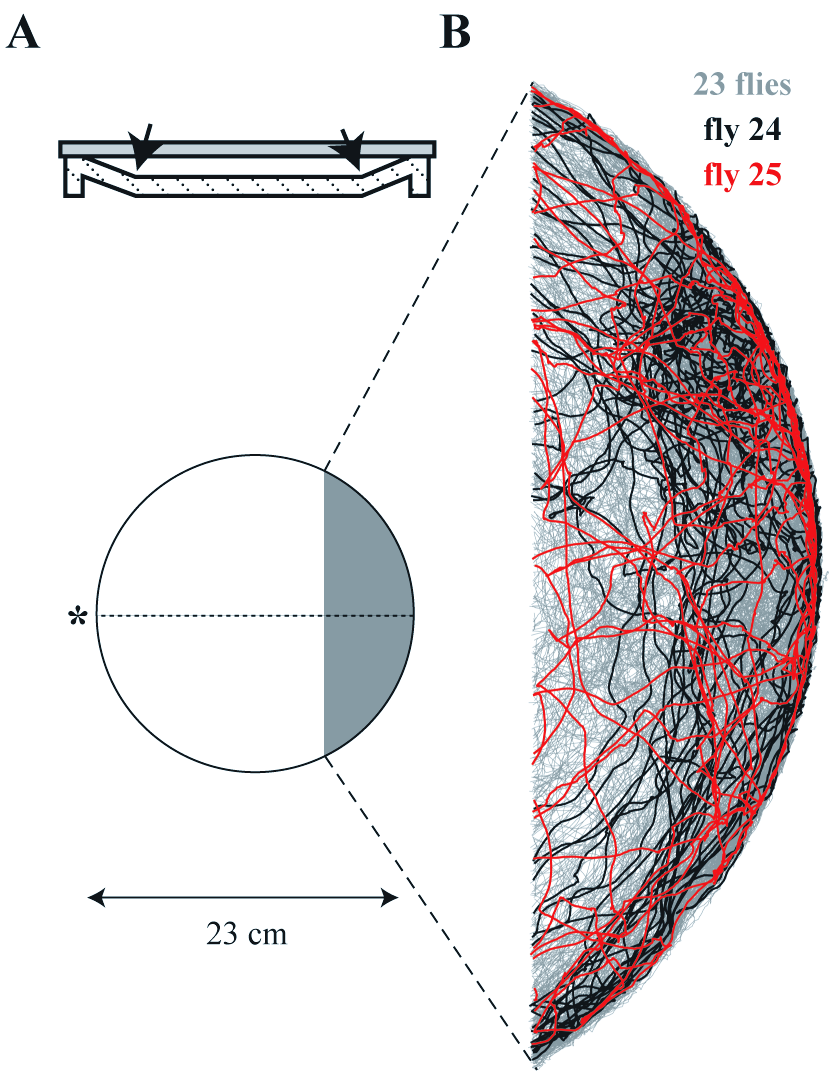

Supplement: Figure S2 — Chambers designed with linear sloped walls are comparable to the chambers designed with sigmoid-linear walls. (A) Side profile of linear slope along denoted cross section (asterisk). Obtuse corners between the wall and floor (arrowheads). (B) Superposition of the individual trajectories for 25 flies (gray) with the trajectories of two flies chosen randomly and highlighted (red and black lines). (3.55 MB TIF) [file pone.0008793.s002.tif]

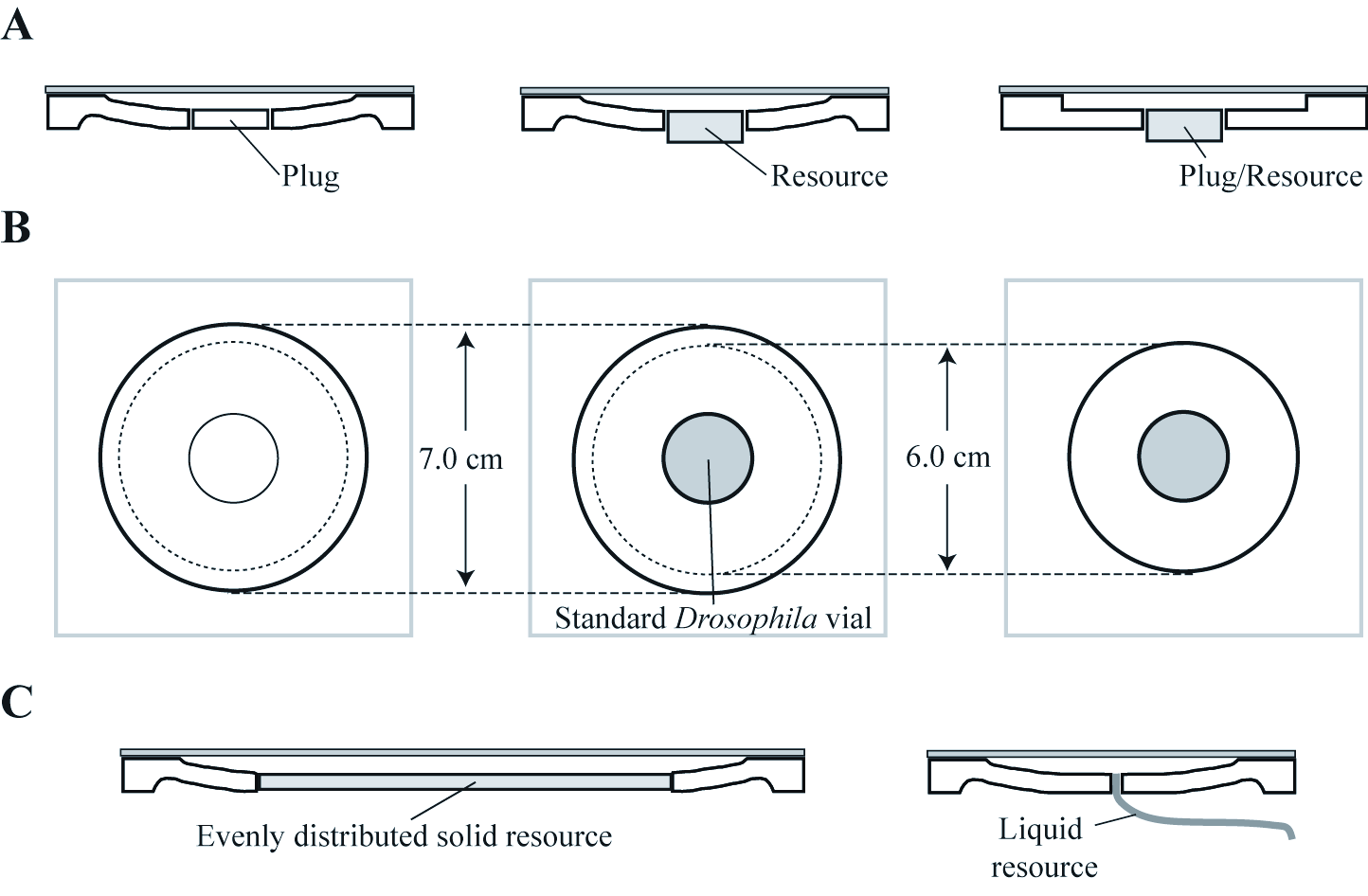

Supplement: Figure S3 — Drawings of various chambers designed for studying social behavior. (A) Side view drawings of chambers designed with a plug used for courtship assays and solid resource used for observations of aggression and the conventional chamber with vertical walls possessing comparable dimensions to chambers with sloped walls. (B) Corresponding top view drawings of chambers shown in A. (C) Alternative chamber designs that could be used for providing an evenly distributed solid resource or a liquid resource from a localized spot. (5.46 MB TIF) [file pone.0008793.s003.tif]
